# Introduction of an Ultraviolet C-Irradiated 4T1 Murine Breast Cancer Whole-Cell Vaccine Model

**DOI:** 10.3390/vaccines11071254

**Published:** 2023-07-18

**Authors:** Gábor J. Szebeni, Róbert Alföldi, Lajos I. Nagy, Patrícia Neuperger, Nikolett Gémes, József Á. Balog, László Tiszlavicz, László G. Puskás

**Affiliations:** 1 Laboratory of Functional Genomics, Biological Research Centre, Temesvári krt. 62, H6726 Szeged, Hungary; neuperger.patricia@brc.hu (P.N.); gemes.nikolett@brc.hu (N.G.); balog.jozsef@brc.hu (J.Á.B.); 2 Department of Physiology, Anatomy and Neuroscience, Faculty of Science and Informatics, University of Szeged, Közép fasor 52, H6726 Szeged, Hungary; 3 CS-Smartlab Devices Ltd., Ady E. u. 14, H7761 Kozármisleny, Hungary; 4 AstridBio Technologies Ltd., Wimmer Fülöp utca 1, H6728 Szeged, Hungary; r.alfoldi@astridbio.com; 5 Avidin Ltd., Alsó Kikötő sor 11/D, H6726 Szeged, Hungary; l.nagy@avidinbiotech.com; 6 Department of Pathology, University of Szeged, Állomás u. 2, H6725 Szeged, Hungary; tiszlavicz.laszlo@med.u-szeged.hu

**Keywords:** 4T1 triple-negative breast cancer, whole-cell vaccine, UVC irradiation, adoptive transfer

## Abstract

The advent of immunotherapy has revolutionized cancer treatments. However, the application of immune checkpoint inhibitors may entail severe side effects, with the risk of therapeutic resistance. The generation of chimeric antigen receptor (CAR) T-cells or CAR-NK cells requires specialized molecular laboratories, is costly, and is difficult to adapt to the rapidly growing number of cancer patients. To provide a simpler but effective immune therapy, a whole-cell tumor vaccine protocol was established based on ultraviolet C (UCV)-irradiated 4T1 triple-negative breast cancer cells. The apoptosis of tumor cells after UVC irradiation was verified using resazurin and Annexin V/propidium iodide flow cytometric assays. Protective immunity was achieved in immunized BALB/c mice, showing partial remission. Adoptive transfer of splenocytes or plasma from the mice in remission showed a protective effect in the naive BALB/c mice that received a living 4T1 tumor cell injection. 4T1-specific IgG antibodies were recorded in the plasma of the mice following immunization with the whole-cell vaccine. Interleukin-2 (IL-2) and oligonucleotide 2006 (ODN2006) adjuvants were used for the transfer of splenocytes from C57BL/6 mice into cyclophosphamide-treated BALB/c mice, resulting in prolonged survival, reduced tumor growth, and remission in 33% of the cases, without the development of the graft-versus-host disease. Our approach offers a simple, cost-effective whole-cell vaccine protocol that can be administered to immunocompetent healthy organisms. The plasma or the adoptive transfer of HLA-matching immunized donor-derived leukocytes could be used as an immune cell therapy for cancer patients.

## 1. Introduction

The complete eradication of solid cancers, such as triple-negative breast cancer (TNBC), remains a challenge. It is widely known that the outgrowth of a tumor reshapes the immune system. A tumor’s evasion of immune clearance may rely on multiple mechanisms, driven by the tumor itself and/or the tumor microenvironment (TME) [1]. The cellular anti-tumor immunity exhibited by CD8 cytotoxic T-cells is hampered by the expression of immunosuppressive mediators, such as PD-L1, CTLA-4, LAG-3, TIM-3, and galectin-1 [2,3,4]. Previously, our group showed the apoptosis of tumor-infiltrating activated T-cells induced by galectin-1 accumulated in the tumor microenvironment [4,5,6]. The generation of tumor-specific antigen (TSA)- or tumor-associated antigen (TAA)-specific T-cells is also restricted by the unresponsiveness or anergy of T-cells in the host. Regulatory T-cells essential for the maintenance of peripheral tolerance in immunohomeostasis have also been widely reported, rendering tumor-specific immune response ineffective in a tumor-bearing host via the production of immunosuppressive cytokines, metabolic reprogramming, and the promotion of metastasis [7,8]. Innate lymphoid cells, such as natural killer (NK) cells mediating cellular cytotoxicity independently of antigen presentation, may also be hampered in cancer. The low infiltration of NK cells into solid tumors has been frequently reported, whereby the extracellular matrix in the TME creates a barrier to infiltration. Metabolic reprogramming, deposition of L-kynurenine, lactate accumulation, and hypoxia may also render NK cells dysfunctional in the TME [9,10]. Among other things, we have previously reported that tumor-induced tolerance is further mediated by the emergence of granulo-monocytopoiesis—the expansion of immature myeloid-derived suppressor cells (MDSCs) with several immunosuppressive functions [11,12]. We previously reviewed therapeutic targeting approaches and features of pro-tumoral inflammatory myeloid cells, which have an inherent immunosuppressive activity and are involved in the promotion of neo-angiogenesis, the mediation of epithelial–mesenchymal transition, and the alteration of cellular metabolism [13]. Other pro-tumoral myeloid cells that may facilitate tumor progression are tumor-associated macrophages (TAMs), with a spectral continuum of alternative polarization states leading to the promotion of tumor growth, immunosuppression, angiogenesis, and cancer cell dissemination [14]. Antigen presentation, a prerequisite for T-cell activation, is also compromised in tumor-bearing hosts via the loss of major histocompatibility complex (MHC) I in tumor cells, the downregulation of MHC II in antigen-presenting cells (dendritic cells or macrophages), and the downregulation of costimulatory molecules, such as CD80, CD86, CD40, and OX40L [15,16,17,18]. The role of B-cells, as antigen-presenting and antibody-producing cells with immunoregulatory functions, is controversial in cancer. Antigen presentation may mediate tumor-cell-specific cellular immunity, while antibody production may directly lead to the elimination of tumor cells via antibody-dependent cellular cytotoxicity (ADCC) or by the activation of the complement system. However, the production of antibodies may lead to the formation of immunocomplexes, which correlates with poor outcomes. Additionally, B-cells may develop into regulatory B-cells which produce IL-10 or TGF-β, mediating the formation of CD4+ regulatory T-cells, instead of anti-tumoral Th2 or Th1 CD4+ T-cell phenotypes [19,20]. 

Several immunotherapy (IT) strategies have been developed to awaken a patient’s immune system and augment anti-cancer effector functions. These ITs have revolutionized cancer treatment, being applied as a monotherapy or to increase the effectiveness of traditional therapies when in combination with radiation therapy or chemotherapy. Among others, we have previously shown the immunomodulatory effects of cisplatin in a 4T1 triple-negative murine breast cancer model, in which low-dose cisplatin could reduce the expansion of MDSCs [21]. The forthcoming small-molecule-based ITs are encouraging due to their good absorption and penetration into tumor tissues, their lower production costs than biologics, and their possible oral bioavailability [13,22,23]. Therapeutic antibodies or antibody derivatives (Fab = fragment antigen-binding region, scFV = single-chain variable fragment, or chimeric antibodies) targeting immune checkpoint modulators are currently part of the clinical routine and have shown great success. The use of immune checkpoint inhibitors (ICIs) began when they were employed as a second-line treatment of PD-1 blockade in lung cancer in 2015 [24,25,26]. Other studies reported higher TCR diversity in cases responding well to PD-1- or CTLA-4-targeting ICI therapies [27,28]. ICIs are beyond the scope of the current study and have been reviewed elsewhere [29,30]. Genetic engineering for the establishment of chimeric antigen receptor T-cells (CAR T-cell) or CAR-NK cells has made the cloning of tumor antigen-reactive T-cell receptor chains into host-derived T-cells or NK cells possible (NK cells can also be irradiated cell lines, e.g., NK92, or umbilical cord blood-derived NK cells). CAR technology has several advantages: (1) avoiding graft-versus-host disease, (2) enabling in vitro expansion, and (3) offering a repeatable treatment tailored to the temporal changes in tumor immunogenicity [31,32,33]. Another approach to augmenting anti-tumor immunity and boosting self-defense mechanisms is vaccination against cancer using a variety of strategies, such as whole-cell vaccine as well as protein-based and nucleic acid-based vaccines. These can be preventative (such as human papilloma virus (HPV) or hepatitis B vaccines) or therapeutic vaccines (e.g., Provenge against prostate cancer using autologous DCs trained to demonstrate antigens of prostatic acid phosphatase) used to combat cancers with an established malignancy [34]. 

Whole tumor cell-based vaccines have recently been reviewed by other scholars, highlighting the advantages of using whole cancer cell-based immunization [35,36,37]. Whole-cell vaccinations demonstrate pan-spectra of tumor antigens inducing multi-clonal expansion of tumor-reactive T-cells, and they may overcome the heterogeneity of tumor cells. The cornerstone of tumor-cell-based vaccines is to attenuate tumor cells to avoid tumor development (X-ray irradiation, freeze–thaw cycles, etc.) and maintain immunogenicity with the preservation of cancer-related epitopes. Furthermore, a common aim among vaccine types is the breakthrough in tumor/TME-induced tolerance against TSA or TAA. The challenges of anti-cancer vaccination are (1) choosing the right technology for a specific cancer type, (2) establishing the delivery platform of the vaccine, and (3) targeting the appropriate TSA or TAA [38,39,40]. Several adjuvants may be administered to boost anti-tumor immunity and increase the effectivity of ITs via the activation of innate immunity, which may facilitate the activation of the adaptive arm of the immune system. Most of the adjuvants of cancer vaccines are developed to activate pattern recognition receptors (such as Toll-like receptors, TLRs) on DCs or macrophages. Adjuvants for cancer vaccine development have recently been reviewed elsewhere [34,41,42,43].

The aforementioned immunosuppressive circumstances may limit the range of T-cell receptors (TCRs) or B-cell receptors available for use in cancer. In line with this assumption, Stonen et al. reported that a naive immune system without cancer-driven tolerance could provide a wider repertoire of TCRs to tumor antigens [44]. Therefore, in our model, we focus on a 4T1 triple-negative breast cancer cell line-based whole-cell vaccine in a naive host, wherein 4T1 cells were UVC irradiated because UVC irradiation was shown to increase the immunogenicity of tumor cells in some studies [45,46,47]. UV irradiation increases the expression of danger signals (such as high-mobility group box 1 and heat shock proteins), as well as antigen presentation on MHC I and exposure of phosphatidyl serine and its uptake by professional antigen-presenting cells, thereby increasing cytotoxic T-cell activation [48]. The adjuvant TLR9 ligand, CpG oligodeoxynucleotide ODN2006, was used in our study to augment both humoral and cellular anti-tumor immunity with the co-application of IL-2 cytokine treatment [49,50]. Our model offers a simple, cost-effective whole-cell vaccine production approach that can be administered to an immunocompetent healthy organism and adoptively transferred to a tumor-bearing host.

## 2. Materials and Methods

### 2.1. Cell Culturing

4T1 cells were purchased from the ATCC (American Type Culture Collection, Manassas, VA, USA) and maintained as described previously [21,51]. Briefly, 4T1 cells were maintained in Roswell Park Memorial Institute 1640 medium (RPMI-1640) with 10% FCS (Euroclone, Milan, Italy). The pH of the cell culture medium was controlled so as to remain between 7.2 and 7.4 prior to use. The medium was supplemented with 2 mM GlutaMAX, 100 U/mL of penicillin, and 100 g/mL of streptomycin (Thermo Fisher scientific, Waltham, MA, USA) before use. Cells were passaged every three days and placed in a humidified incubator at 37 °C with 5% CO_2_ (Sanyo, Osaka, Japan).

### 2.2. Flow Cytometric MHCI Investigation

4T1 cells were grown in the logarithmic phase on 10 mm wide tissue culture Petri dishes (Corning Life Sciences, Tewksbury, MA, USA), the supernatant was removed, and the cells were detached by the addition of 5 mL of Accutase (Thermo Fisher scientific). The cells were washed with 10 mL of PBS (Merck, Darmstadt, Germany) and centrifuged at 350 g at RT for 5 min. Antibodies were diluted in IFB (immunefluorescence buffer, PBS with 2% FCS) to the following levels: anti-mouse MHCI H-2K^d^ Alexa Fluor^TM^ 488 1:200 dilution (Biolegend, San Diego, CA, USA, clone SF-1.1, cat. numer 116609), and anti-mouse MHCI H-2K^b^ Alexa Fluor^TM^ 488 1:200 dilution (Biolegend, clone AF6-88.5, cat. number 116510). The 4T1 cells (2.5 × 10^5^) were incubated with the antibodies in 100 µL of IFB in separate tubes or left unstained for 30 min at RT. The cells were washed with 1 mL of PBS and centrifuged at 350× *g* at RT for 5 min. The cells were resuspended in 250 µL of IFB and assessed using a FACSCalibur (Becton Dickinson, Franklin Lakes, NJ, USA). The samples were analyzed using CellQuest^TM^ Pro v5.1 (Becton Dickinson).

### 2.3. Whole-Cell Vaccine Generation

4T1 cells were cultured in the logarithmic phase to 70% confluency on a 10 mm wide tissue culture Petri dish (Corning Life Sciences). The supernatant was aspirated, and the cells were washed with 5 mL of PBS (Merck) and detached using 3 mL of Accutase (Thermo Fisher scientific) at room temperature for 5 min. The cells were harvested by adding 7 mL of PBS and counted using a Bürker chamber and trypan blue excision dye (Merck). The cells (10^6^/mice + extra 10%) were centrifuged at 1500 rpm for 5 min, before being resuspended in 10 mL of PBS and pipetted into a 10 mm wide Petri dish. The cells were placed into an irradiator with the Petri dish uncovered to avoid ultraviolet light being filtered out by the plastic. UVC irradiation was carried out using an Ultra Lum UVC-508 (Ultra Lum, Inc., Clearwater, FL, USA) with four G8T5 Sankyo UVC lamps (Sankyo, Tokyo, Japan) for 2 × 5 min, which resulted in a 42,857 J/m^2^ UVC dose. The cover of the Petri dish was removed when it was inside the UVC irradiator, which had a germicide effect and rendered it sterile. Additionally, the UVC irradiator was placed under a sterile hood. The cells were gently moved after the first 5 min of irradiation from the wall to the center to ensure a homogenous dose. The cells were further processed immediately and injected into the mice without the occurrence of septic shock or any sign of microbial contamination. The cells were harvested by pipetting, and were counted and centrifuged at 1500 rpm for 5 min. The cells were resuspended in FCS-free RPMI at a concentration of 10^7^/mL. The BALB/c mice were immunized with 10^6^ UVC-irradiated 4T1 cells on days 0, 7, and 21 via injection with 25 µL each into the left and right footpads and 25 µL each into the left and right flanks, for a total volume of 100 µL. The control (sham) group received only FCS-free RPMI injections to mimic immunization.

### 2.4. Resazurin Viability Assay

The viability of the UVC-irradiated 4T1 cells was measured using the resazurin assay, as described previously [52,53]. Briefly, the 4T1 cells (8 × 10^3^ cells/well) were seeded into 96-well plates (Corning) in RPMI media containing 10% FCS and incubated overnight. UVC irradiation was carried out using an Ultra Lum UVC-508 with four G8T5 Sankyo UVC lamps for 2 × 5 min, which resulted in a 42,857 J/m^2^ UVC dose. The cells were shaken gently after the first 5 min of irradiation. The plate cover was removed during UVC irradiation. The cells were assayed after 72 h of incubation. The control, untreated cells were handled in the same way but without irradiation. Resazurin reagent (Sigma–Aldrich, Budapest, Hungary) was added at a final concentration of 25 μg/mL. After 2 h of incubation at 37 °C with 5% CO_2_, fluorescence (530 nm excitation/580 nm emission) was recorded using a multimode microplate reader (Cytofluor4000, PerSeptive Biosytems, Framingham, MA, USA). The fluorescence values of 3 replicates in relation to their viability were visualized using GraphPad Prism^®^ 5 (La Jolla, CA, USA).

### 2.5. Flow Cytometric Viability Assay

The viability of the UVC-irradiated 4T1 cells was measured using an AnnexinV/propidium iodide flow cytometric assay, as described previously [51,54]. Briefly, 4T1 cells were cultured and detached with Accutase as described above; they were then counted, and 5 × 10^6^ cells were plated into a 10 mm Petri dish containing a 10^6^/mL concentration of PBS. The cells were placed into an irradiator without the Petri dish cover. UVC irradiation was carried out using an Ultra Lum UVC-508 with four G8T5 Sankyo UVC lamps for 2 × 5 min, which resulted in a 42,857 J/m^2^ UVC dose. The untreated control cells were handled in the same way but without irradiation. The cells were shaken gently after the first 5 min of irradiation. The cells were harvested by pipetting, and then counted and centrifuged at 1500 rpm 5 min. The cells were resuspended in Annexin V binding buffer (ABB; 0.01 M HEPES, 0.14 M NaCl and 2.5 mM CaCl_2_, pH 7.4, Merck) at a 4 × 10^6^/mL concentration. Then, 2 × 10^5^ cells in 48.75 µL of ABB were stained with Annexin V-Alexa Fluor^TM^ 488 (1.25:50, Thermo Fisher Scientific) for 15 min in the dark at room temperature. In order to dilute (5×) Annexin V-Alexa Fluor^TM^ 488, 10 μg/mL of propidium iodide (PI, Merck) mixed with 200 μL of ABB was added to the cell suspensions directly before acquisition. In total, 10,000 events per sample were detected using a FACSCalibur cytofluorimeter and data analyses were performed using the CellQuest^TM^ software. Early (Annv+/PI−) and late (AnnV+/PI+) apoptotic cells were gated, and data from 3 replicates were visualized using GraphPad Prism^®^ 5 (La Jolla, CA, USA).

### 2.6. Establishment of the 4T1 Tumor Model and Ethical License

The animal experiments were performed in accordance with the animal experimentation and ethics guidelines of the EU (2010/63/EU). The experimental protocols were approved by the responsible governmental agency (National Food Chain Safety Office) in possession of the ethical clearance XXIX./128/2013. Female Charles River-derivative BALB/c mice (8–10-week-old) were purchased from Kobay Ltd. (Ankara, Turkey) and were injected orthotopically with 4T1 breast carcinoma cells (5 × 10^4^ cells), as described previously [55]. The animals had free access to food and water. Six mice were included in each experimental group. Tumors were evaluated macroscopically with the following parameters: (1) the incidence of palpable tumors was determined through daily monitoring of the animals in each experimental group; (2) tumor size was measured using a precision caliper and calculated according to the formula d^2^ × D × 0.5, where d and D are the minor and major diameters, respectively; and (3) after euthanizing the animals, the weights of the lungs were measured. Bone marrow, spleen, and blood were processed in order to isolate fresh cells or plasma. Mice showing signs of suffering (loss of 15% of body weight and/or loss of the righting reflex and/or unable to eat and drink) were sacrificed due to (ethical) legislation.

### 2.7. Immunohistochemistry (IHC)

The immunohistochemistry analysis was performed as described previously by our laboratory with some modifications [56,57]. Briefly, the mice were sacrificed, and 2 mm carcinoma-like tissue pieces were excised and placed in 3.7% formaldehyde (37% formaldehyde was diluted 10× with PBS, Molar Chemicals, Budapest, Hungary). The tissues were fixed overnight, then embedded in paraffin, and cut into 4 μm sections. Immunohistochemistry was performed using an EnVision FLEX Mini Kit (DAKO, part of Agilent Technologies Inc., Santa Clara, CA, USA). Antigen retrieval was performed using a PT Link machine in citrate buffer at a pH of 6.0 (DAKO). The primary antibodies used for immuno-histochemistry were broad-spectrum anti-pan-cytokeratin in a 1:100 dilution (clone KL1, cat. number: 10042, Histopathology Ltd., Pécs, Hungary) and anti-vimentin in a 1:200 dilution (cat. number: RM-9120, Labvision Ltd., Värmdö, Sveden), which were incubated overnight. The secondary antibody cocktail containing polyclonal goat anti-rabbit-HRP and anti-mouse-HRP (DAKO, K8000) was incubated for 30 min. Visualization was undertaken using an EnVision FLEX DAB+ Chromogen System (DAKO, GV825). Hematoxylin staining was applied on a consecutive tissue section. After eosin counterstaining of the IHC for 5 min, the slides were mounted and scanned using a Pannoramic Digital Slide Scanner (3D Histech Ltd., Budapest, Hungary).

### 2.8. Adoptive Transfer Experiments

The mice were sacrificed by CO_2_ inhalation, and bone marrow cells (BMs), splenocytes, and plasma were freshly isolated using sterile preparation. First, the withdrawal of blood was performed via puncturing the heart with a 1 mL syringe (26G needle, B. Braun, Melsungen, Germany) containing 50 µL of 30mg/mL Na_2_EDTA (Merck) solution. Blood was centrifuged at 3000 rpm for 10 min, and plasma was harvested, aliquoted in 110 µL, and frozen at −80 °C. For the isolation of splenocytes, spleen removal was performed; the spleens were smashed on a 70 µm cell strainer (Merck) above a 50 mL centrifuge tube. The cells were washed with PBS and centrifuged at 1500 rpm for 5 min. Red blood cells were lysed via incubation with 5 mL of ACK solution (0.155 M NH_4_Cl, 10 mM KHCO_3_, 0.1 mM Na_2_EDTA, pH 7.3, Merck) at room temperature for 5 min. A total of 10 mL of complete RPMI was added, and the cells were filtrated again on a 70 µm cell strainer. The cells were counted using a Bürker chamber and centrifuged at 1500 rpm for 5 min. The cells were gently dissolved in a freezing medium (90% FCS + 10% DMSO, Merck), placed in a cryobox at −80 °C for 48 h, and then placed in a liquid nitrogen tank for long-term storage. For the isolation of BM cells, femurs and tibias were excised and cleaned of muscles, and epiphysis was cut. A 1 mL syringe filled with 1 mL of PBS was inserted into the lumen and BM was washed in a 50 mL centrifuge tube. The BM samples of the bones of one mouse were pooled. The isolate was suspended with PBS and then centrifuged at 1500 rpm for 5 min. Red blood cells were lysed using 5 mL of ACK, as described previously. The cells were washed with 10 mL of complete RPMI, counted, and centrifuged at 1500 rpm for 5 min. The cells were gently dissolved in a freezing medium and handled as previously described for splenocytes. The adoptive transfer of BM, splenocytes, or plasma is described in detail in the corresponding figure’s legend for better clarity.

### 2.9. Detection of Anti-4T1 IgG Antibodies

4T1 cells were cultured in the logarithmic phase to 70% confluency on a tissue culture Petri dish. The supernatant was aspirated, and the cells were washed with 5 mL of PBS (Merck) and detached using 3 mL of Accutase (Thermo Fisher Scientific) at room temperature for 5 min. The cells were harvested by adding 7 mL of PBS and then counted using a Bürker chamber and trypan blue excision dye (Merck). In total, 2 × 10^5^ 4T1 cells were incubated with plasma and then 5× diluted in 100 µL of IFB at 4 °C for 1 h. The cells were washed with 1 mL of IFB and centrifuged at 1500 rpm for 5 min. The cells were resuspended in 100 µL of IFB containing goat-anti-mouse anti-IgG Alexa Fluor^TM^ 488 (Thermo Fisher Scientific, A-11001 400× diluted), and then incubated at 4 °C for 30 min. The cells were washed with 1 mL of IFB and then centrifuged at 1500 rpm for 5 min. The cells were resuspended in 300 µL of IFB, and 10 µg/mL of propidium iodide was added (3 µL from 1 mg/mL stock) right before acquisition to gate on viable cells. In total, 10,000 events per sample were detected using an FACSCalibur cytofluorimeter, and data analyses were performed using the CellQuest^TM^ software.

### 2.10. Statistics

Statistical analysis was performed using Student’s *t*-test to evaluate statistical significance (set at * *p* < 0.05, ** *p* < 0.01, and *** *p* < 0.001) between two given experimental groups.

## 3. Results

### 3.1. UVC Irradiation of 4T1 Cells Used to Prepare a Whole-Cell Vaccine

**Figure 1:**
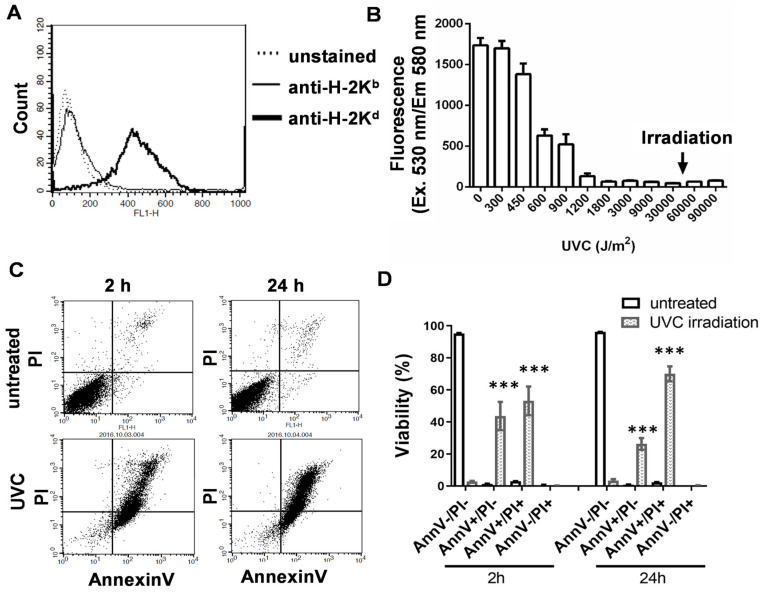
UVC-irradiated 4T1 cell-based whole-cell vaccine based on apoptotic 4T1 cells. (**A**) The cell surface expression of the BALB/c-specific MHCI H-2K^d^ haplotype (black line) on 4T1 cells was investigated via flow cytometry. UVC irradiation was carried out twice for 5 min using an Ultra Lum UVC-508 with four G8T5 Sankyo UVC lamps, which resulted in a 42,857 J/m^2^ UVC dose. (**B**) The viability of the UVC-irradiated 4T1 cells was assayed using the resazurin assay after 72 h. (**C**) Representative flow cytometry dot plots showing the viability of UVC-irradiated 4T1 cells, as assayed by AnnexinV (FL1)/PI(FL3) staining after 2 h or 24 h. (**D**) The viability of UVC-irradiated 4T1 cells demonstrating early apoptotic (AnnV+/PI−) and late apoptotic cells (AnnV+/PI+) at 2 h or 24 h after irradiation. Pairwise comparison was performed between the corresponding untreated and UVC-irradiated samples, *** *p* < 0.001.

The triple-negative 4T1 mouse breast cancer cells were assessed regarding the expression of MHC I as a prerequisite for the presentation of tumor-associated or tumor specific-antigens. The flow cytometry analysis showed H-2K^d^ MHCI haplotype expression characteristic of BALB/c on the surfaces of the 4T1 cells (Figure 1A). The unrelated C57BL/6 strain-specific anti-H-2K^b^ was used as a control, and unstained cells were used as controls to set the autofluorescence (Figure 1A). UVC irradiation was used because of its known effects, including (1) increasing antigen exposition, (2) increasing the expression and/or release of endogenous danger signals (such as ATP, cytochrom C, or heat shock proteins), and (3) ability to render 4T1 cells apoptotic in order to avoid tumor initiation. The 4T1 cells were cultured in a logarithmic phase to 70% confluency and detached using Accutase in order to maintain cell surface-related structures as much as possible, as well as to avoid the digestion of epitopes by the alternative trypsin detaching solution. The lethal dose of UVC was titrated, and the 4T1 cells were subjected twice to 5 min of irradiation in an Ultra-Sum UVC cross-linker machine equipped with four G8T5 Sankyo UVC lamps that yielded a 42,857 J/m^2^ UVC dose. The resazurin viability assay showed the effects of UVC with saturation over the sub-lethal dose (Figure 1B). Next, flow cytometry was used to detect early apoptotic AnnV+/PI− cells and late apoptotic cells (AnnV+/PI+) after 2 h or 24 h of irradiation (Figure 1C). The percentage of early apoptotic cells (mean ± SD) following irradiation was 43.7 ± 8.8% versus 1.2 ± 0.2% when untreated, and the percentage of late apoptotic cells was 53.1 ± 8.1% vs. 2.9 ± 0.1% when untreated after 2 h of UVC irradiation. The percentage of early apoptotic cells was 26.3 ± 3.6% vs. 0.9 ± 0.05% when untreated, and the percentage of late apoptotic cells was 70 ± 4.6% vs. 2.3 ± 0.1% when untreated after 24 h of UVC irradiation (Figure 1D).

### 3.2. Vaccination with UVC-Irradiated 4T1 Whole-Cell Vaccine Resulted in Partial Remission and Lower Lung Metastatic Burden

**Figure 2:**
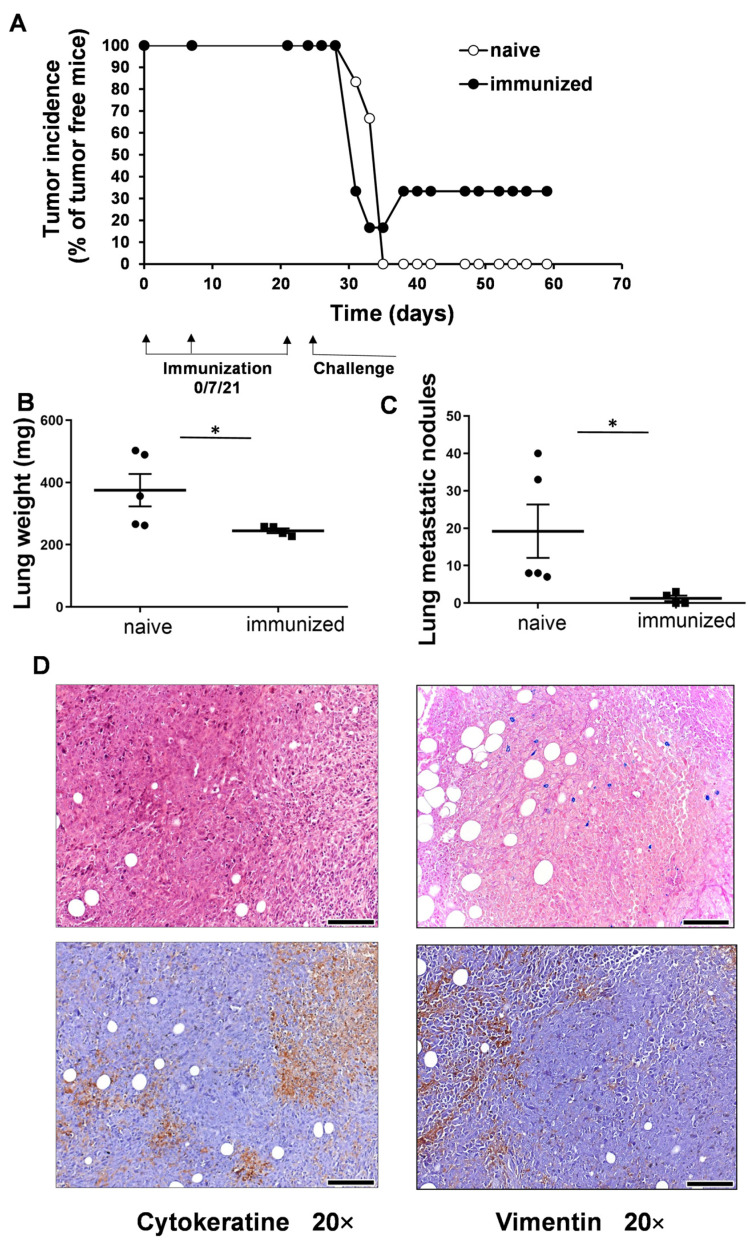
The immunization of BALB/c mice with UVC-irradiated 4T1 cells resulted in the partial remission of breast cancer. (**A**) The BALB/c mice were immunized with 10^6^ UVC-irradiated 4T1 cells intradermally on days 0, 7, and 21 after injection with 25 µL each into the left and right footpads and 25 µL each into the left and right flanks, for a total volume of 100 µL. The control (sham) group received only FCS-free RPMI injections to mimic immunization. The challenge was induced by the injection of 5 × 10^4^ 4T1 living cells orthotopically into the mammary gland (n = 6 mice/group) on the 24th day. Tumor incidence was monitored regularly. Two of the six animals in the immunized group showed remission without palpable solid tumors by the end of the experiment. (**B**,**C**) The lung weights and numbers of macroscopic metastatic nodules on the lungs are lower in the immunized group. Pairwise comparison was performed between the corresponding naive (circle) and immunized (square) samples, * *p* < 0.05. (**D**) Following the euthanization of the animals, necropsy identified microscopic carcinoma in the mammary glands of those with remission, and IHC showed pan-cytokeratin and vimentin-positive 4T1 breast cancer cells (brown residue) surrounded by tumor stroma (non-reactive area). Hematoxylin staining was applied on a consecutive tissue section (upper panel). The scale bar is 100 µm.

The female BALB/c mice were immunized with the UVC-irradiated 4T1 whole-cell vaccine three times before being challenged with the injection of 5 × 10^4^ 4T1 cells orthotopically into the mammary gland. The control group was injected only with the FCS-free RPMI vehicle. Here, 100% of the naive control mice developed 4T1 tumors, while tumor incidence was 66.6% in the vaccinated group, by the 59th day of the experiment. One mouse in the immunized group became free of palpable tumors by the 34th day of the experiment, and one mouse did not develop any tumors (Figure 2A). Cells of 4T1 breast cancer frequently metastasize the lungs, and so the weights of the lungs and the numbers of macroscopic metastatic nodules on the lungs were monitored. The weights of the lungs (mean ± SD) were 244.9 ± 15.2 mg in the vaccinated group vs. 375.2 ± 116 mg in the control group (Figure 2B). The numbers of metastatic nodules (mean ± SD) were 1.2 ± 1.5 in the immunized group vs. 19.2 ± 15.9 in the control group (Figure 2C). The necropsy of a mouse showing remission in palpable tumors showed a 2 mm diameter carcinoma, like a bulb of tissue, that was stained for pan-cytokeratin and vimentin via immunohistochemistry, and verified as breast carcinoma (Figure 2D). The positivity of 4T1 cells to pan-cytokeratin and vimentin has been shown previously [58,59].

### 3.3. Adoptive Transfer of Plasma or Splenocytes from Mice with Remission Endowed the Recipient Mice with Protective Immunity against Living 4T1 Cells

**Figure 3:**
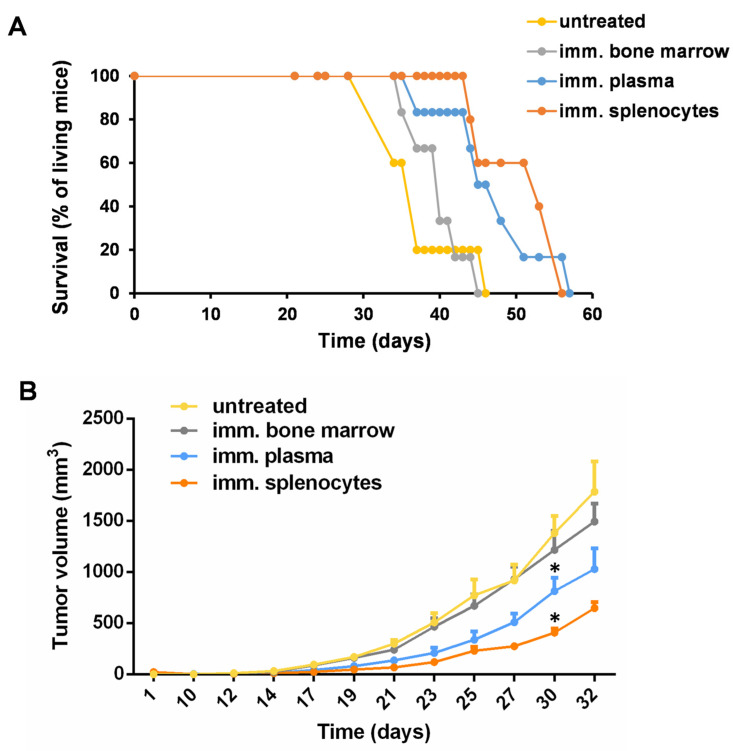
Adoptive transfer of bone marrow cells, splenocytes, or plasma from immunized mice with remission into naive BALB/c mice receiving living 4T1 cells. The two 4T1-immunized animals with remission were challenged with 1 × 10^6^ Accutase-detached and UVC-irradiated 4T1 cells on the 56th day. On the same day, 24 naive BALB/c mice were injected with 5 × 10^4^ living 4T1 cells into the mammary gland orthotopically. After three days (59th day), the animals with remission that had been re-challenged were euthanized, and bone marrow (BM), splenocytes, and plasma were isolated for adoptive transfer. Four groups were created (n = 6 mice/group) and were treated with 2 × 10^6^ BM, or 5 × 10^6^ splenocytes, or 100 µL of plasma intravenously, or left untreated. (**A**) Survival of the animals and (**B**) tumor growth were monitored daily. Pairwise comparison was performed between the corresponding untreated and immunized samples, * *p* < 0.05.

The two mice without palpable tumors in the immunized group were challenged with 1 × 10^6^ Accutase-detached and UVC-irradiated 4T1 cells on the 56th day of the experiment. Three days later, biological samples, such as bone marrow cells, plasma, and splenocytes, were isolated from the mice showing remission in the development of palpable 4T1 tumors. Naive female BALB/c mice were injected with 5 × 10^4^ 4T1 cells into the mammary gland. Adoptive transfer (AT) was carried out from the mice with remission into the naive BALB/c mice, the latter of which received living 4T1 cells three days before the AT. The AT involved a single injection of 2 × 10^6^ BM, 5 × 10^6^ splenocytes, or 100 µL of plasma intravenously. The untreated control mice received only a vehicle of FCS-free RPMI. On the 43rd day, the splenocyte or plasma transfers prolonged the survival of 83% of the mice, compared to a 33% survival rate in the control group. On the 54th day, all control mice were lost, while splenocyte or plasma transfers resulted in 33% or 16% of the treated mice being free of tumors, respectively (Figure 3A). Tumor volume was regularly measured using a caliper, and the volume of the spheroid was calculated. The individual tumor volume values are listed in Supplementary Figure S1. Splenocyte transfer slowed down the progression of 4T1 carcinoma development (mean ± SD), yielding tumors with a size of 647 ± 135 mm^3^ versus 1784 ± 665 mm^3^ in the control group on the 32nd day. The plasma transfer slowed down the progression of the tumor to 1028 ± 495 versus 1784 ± 665 mm^3^ in the control group on the 32nd day (Figure 3B). The bone marrow transplant did not have a protective effect, possibly because of the excess of immature leukocyte precursors.

### 3.4. The UVC-Irradiated 4T1 Whole-Cell Vaccine Resulted in the Production of 4T1-Binding IgG Antibodies 

**Figure 4:**
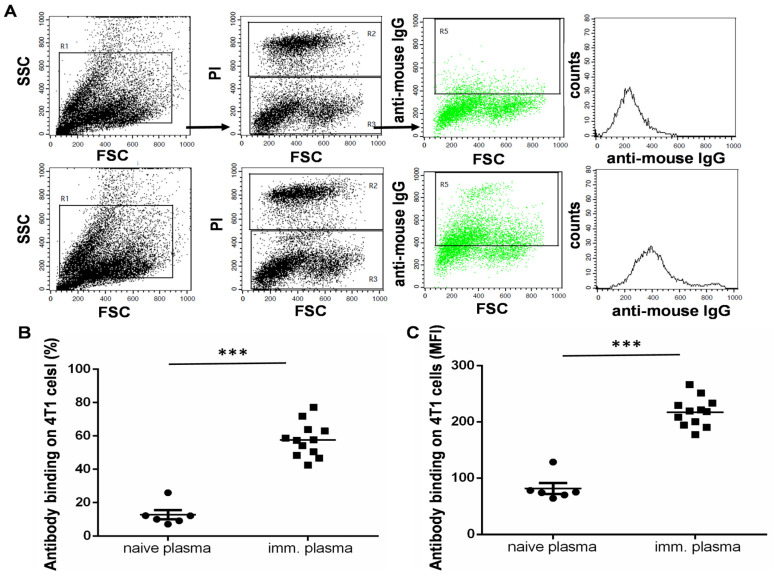
The humoral response of 4T1-whole-cell-vaccinated C57BL/6 animals. The mice were immunized with 1 × 10^6^ Accutase-detached and UVC-irradiated 4T1 cells on days 0, 7, and 21, as described previously. ODN2006 adjuvant (10 µg/mL in 100 µL) was applied intraperitoneally in parallel with each immunization. On the 24th day, the animals were sacrificed, and the plasma of the immunized C57BL/6 mice was isolated. (**A**) Representative flow cytometry dot plots following the incubation of living Accutase-detached 4T1 cells with the 5× diluted plasma of the immunized C57BL/6 mice. The cells were washed and labeled with anti-mouse IgG Alexa Fluor **^TM^** 488 secondary antibody. PI was added to gate PI-negative (R3) living 4T1 cells (upper raw = control, non-immunized samples, and lower raw = immunized samples). The R1 gate was used to exclude cell debris and cell aggregates, the R3 gate was used to analyze PI-negative living cells within the R1, and the R5 gate was applied to analyze seropositive cells within the R3 living population. (**B**) The percentage or (**C**) median fluorescence intensity (MFI) of seropositivity, showing an increase in 4T1 cell surface epitope-binding IgG antibodies in the immunized group. A pairwise comparison was performed between the naïve and immunized samples, *** *p* < 0.001.

Humoral immunity was measured by examining the production of IgG antibodies with binding of the Accutase-detached 4T1 cell line. Plasma was isolated from the immunized mice and assayed using flow cytometry with the incubation of living 4T1 cells freshly detached by Accutase; following incubation and washing, anti-mouse IgG Alexa Fluor 488 was added. Propidium iodide was added to gate out dead cells to exclude non-specific binding and to analyze only live 4T1 cells which are capable of binding IgG antibodies from the plasma of the immunized mice (Figure 4A). Both parameters, the percentage of seropositive 4T1 cells and the median fluorescence intensity (MFI) values, showed a significant increase in humoral immunity in the vaccinated mice (Figure 4B,C).

### 3.5. Adoptive Transfer of Plasma or Splenocytes from the UVC-Irradiated 4T1-Whole-Cell-Vaccinated C57BL/6 Mice into Cyclophosphamide Pre-Treated Non-Syngeneic BALB/c Mice

**Figure 5:**
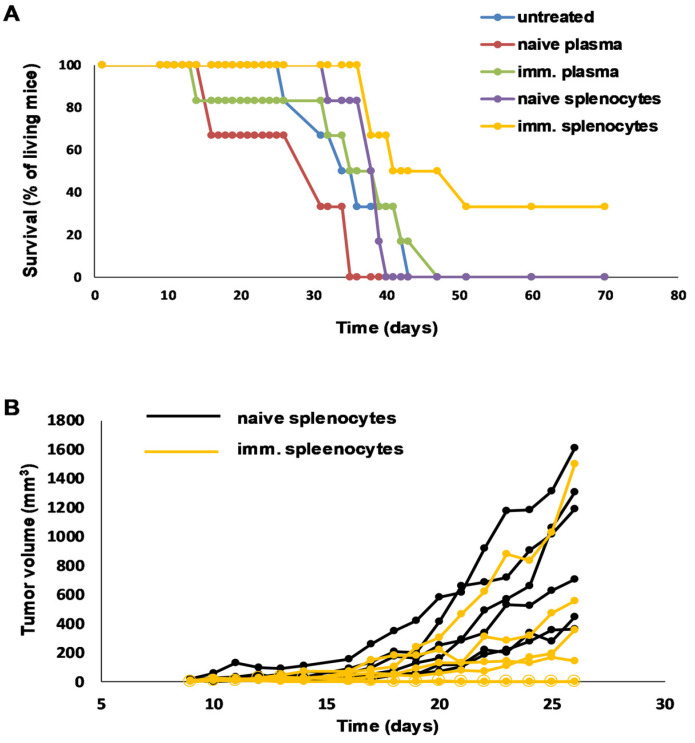
Adoptive transfer of plasma or splenocytes from UVC-irradiated 4T1-immunized C57BL/6 mice into cyclophosphamide-treated recipient BALB/c mice. The C57BL/6 mice were immunized with an Accutase-detached and UVC-irradiated 4T1 whole-cell vaccine as described previously. The recipient BALB/c mice were injected with 1 × 10^4^ living 4T1 cells orthotopically into the mammary gland. On the 11th day, the recipient BALB/c mice were prepared for the injection of a splenocyte graft by giving them 100 mg/kg body weight cyclophosphamide i.p. After 5 h, the recipient BALB/c mice were treated with naive or immunized plasma that was twice diluted in physiologic salt at a volume of 100 µL; 3 × 10^7^ naive or immunized splenocytes were injected twice in the first week and 6 × 10^6^ splenocytes were injected twice in the second week, with 3-day intervals. The blood plasma was heat inactivated at 56 °C for 45 min to inactivate the complementary system. Additionally, IL-2 (0.8 µg/mice) and ODN2006 (1 µg/mice) in 100 µL i.p. were combined with each adoptive transfer. (**A**) The survival of the mice and the (**B**) tumor growth were monitored regularly.

Living 4T1 cells were injected into the mammary gland of the BALB/c mice on the starting day of the experiment to establish a TNBC murine model. In order to mimic the non-syngeneic differences among human individuals bearing different HLA alleles, the adoptive transfer was carried out from UVC-irradiated 4T1-whole-cell-vaccinated C57BL/6 mice into non-immunocompatible BALB/c mice. Vaccination was carried out as described above; plasma and living splenocytes were preserved at −80 °C and in liquid nitrogen, respectively. After 11 days, the recipient BALB/c mice rendered for AT of splenocytes were pre-treated with cyclophosphamide to prevent host-versus-graft and graft-versus-host diseases. After 5 h of cyclophosphamide treatment, the AT started. In order to augment the effects of AT, IL-2 and ODN2006 adjuvants were injected i.p. into the recipient mice simultaneously with AT of plasma or splenocytes. On the 36th day following tumor cell transplantation, 100% of the recipient animals were alive after receiving AT of splenocytes, 83% were alive after receiving naive splenocytes, 50% were alive after receiving AT of plasma, and none of the mice that had received naive plasma were alive. In total, 33% of the untreated control mice were alive 36 days after transplantation (Figure 5A). These untreated control mice were of BALB/c origin and received only vehicle FCS-free RPMI cell culture media with the injection of living 4T1 tumor cells. On the 70th day following tumor cell transplantation, 33% of the recipient animals that had received AT of splenocytes were alive, and no mice from the other groups were alive on the 70th day of the experiment (Figure 5A). The incidence rates of 4T1 tumors were 100–100% in the mice receiving both naive splenocytes and immunized mice-derived splenocytes via injection by the 21st day of the experiment. The remission of palpable tumors occurred on the 22nd day, and the remission of other palpable tumors occurred on the 23rd day after the injection of living 4T1 cells in the group receiving AT of splenocytes from the immunized C57BL/6 mice as a possible mode of graft-mediated tumor clearance. The individual tumor growth kinetics are plotted in Figure 5B. Two of the six mice receiving AT of splenocytes from the immunized mice showed remission, and another two mice showed reduced tumor growth kinetics compared to the control mice injected with naive splenocytes. The statistical significance of tumor growth was only achieved on the 24th day of the experiment. A pairwise comparison was performed between the corresponding untreated and immunized samples, * *p* < 0.05.

## 4. Discussion

Immunoediting drives a cancer’s microevolution to help it escape immunosurveillance and, thus, shapes the immune system’s responsivity to tumor antigens. In an immunosuppressive tumor-bearing host, the augmentation of the immune defense mechanisms, such as phagocytosis, antigen presentation, and the formation of an activating immunological synapse, which lead to effective cellular and humoral responses, should be facilitated by ITs. At present, a series of ITs are available or are in clinical trials, such as small-molecule-based ITs, ICIs, whole-cell cancer vaccines, and protein- or nucleic acid-based cancer vaccines. In our model, we focused on a UVC-irradiated murine 4T1 TNBC cell line-based whole-cell vaccine model. We showed MHCI H-2K^d^ expression on the surface of 4T1 cells as a prerequisite, demonstrating TSA or TAA to cytotoxic CD8+ T-cells. To avoid the outgrowth of breast cancer in the syngeneic BALB/c mice, BALB/c-derived 4T1 cells were lethally irradiated with UVC. We confirmed the lethal dose of the applied UVC using resazurin and AnnV/PI viability assays. The 4T1 cells in the whole-cell vaccine underwent apoptosis as early as 2 h after UVC irradiation. The vaccination of the BALB/c mice resulted in remission in 33% of the cases, with a decreased metastatic burden in the lungs. One mouse with remission showed shrinkage in the palpable tumor after necropsy, and pan-cytokeratin (reactivity with 1, 2, 5, 6, 7, 8, 10, 11, 14,17, 18 and 19 cytokeratins) and vimentin IHC staining verified the presence of carcinoma, which was under the control of the immune system. In order to verify that the elimination of the palpable tumor was due to effective clearance, adoptive cell transfer and plasma transfer were carried out in the naive BALB/c mice. Hicks et al. reported the use of BALB/c mice with spontaneous regression/complete resistance to the challenge of living tumor cells to isolate splenocytes as a graft to naive hosts, with successful elimination of S180 tumor cells in wild-type BALB/c mice [60]. In our experiments, the mice showing remission were challenged with UVC-irradiated 4T1 cells to boost reactive T- and B-cells. After three days, the naive mice were injected with living 4T1 cells and adoptively transferred with splenocytes, bone marrow cells, and plasma from the immunized mice with remission. The tumor growth and survival of the recipient mice were monitored daily. The strongest anti-tumor effect was found for a single injection of splenocyte graft, and the second most effective treatment was the convalescent plasma treatment. Carmi et al. showed that allogeneic IgG-coated tumor cells were engulfed by DCs, and these primed DCs induced a potent tumor-specific T-cell response [61]. As such, the effectiveness of the convalescent plasma treatment may depend on increased phagocytosis and antigen presentation. Another mechanism explaining the effectivity of the plasma treatment could be NK cell-mediated ADCC [62]. 

We did not perform selective depletion of leukocytes of the spleen because other studies previously showed that macrophages, granulocytes, NK cells, T-cells, and B-cells may cooperatively contribute to the protective effect [60,63,64]. Next, we sought to model the adoptive transfer of mismatched MHC from the immunized C57BL/6 mice into the BALB/c recipient mice; because humans are not syngeneic, this experimental setup can model HLA haplotype differences between human subjects. Cyclophosphamide pre-treatment was shown to enhance the effectivity of AT of splenocytes from immunized mice to a host, the localization of adoptively transferred lymphocytes, and the proliferation of tumor-reactive donor-derived T- and B-cells; the cytokine milieu in the host was positively influenced by lymphodepletion. Cyclophosphamide was also reported to suppress the host-versus-graft disease after AT [65]. Cyclophosphamide was also reported as an immunomodulator that supports the generation of anti-tumor T-cell responses [66]. Cyclophosphamide administration was reported by Luznik et al. to support immunotherapies, with the option of adoptive transfer from HLA-mismatched donors [67]. In our model, the pre-treatment of the BALB/c mice with MHC-I H-2K^d^ alleles using cyclophosphamide enabled us to avoid the rejection of adoptively transferred splenocytes from the immunized C57BL/6 mice with MHCI H-2K^b^ alleles. Interleukin-2 treatment was used in our study to increase the activation of allogeneic T-cells. IL-2 administration was shown to improve tumor ITs by improving tumor-specific T-cell-mediated cellular response [68,69]. Schwaab et al. showed a 50% overall objective response rate in metastatic renal cell carcinoma treated with autologous tumor lysate-primed DCs plus IL-2 therapy [70]. Additionally, a 50% overall response rate was observed in cyclophosphamide lymphodepleted stage IV melanoma patients receiving autologous MART-1 (melanoma-associated antigen recognized by T-cells), peptide-pulsed DCs, and ex vivo reactivated tumor-infiltrating lymphocytes plus IL-2 treatment [71]. IL-2 administration was also reported to increase the effectivity of autologous osteosarcoma-cell-derived vaccination in dogs [72]. We used an adjuvant, the TLR9 receptor agonist ODN2006 with unmethylated CpG motifs, and showed that it can activate B-cells, macrophages, and myeloid and plasmacytoid DCs [73]. 

In our study, 33% of the mice were still alive 70 days after the adoptive transfer, while 100% of the control, untreated (non-immunized but challenged with living 4T1 cells) animals had died. The growth of tumor was slowed in the vaccinated group, as two mice showed complete remission and two developed tumors at a slower rate than the untreated control mice. Although 4T1 cell surface epitope-recognizing IgG antibodies were found in the immunized C57BL/6 plasma samples, plasma transfer showed a moderate effect: 50% were alive after AT of plasma on the 36th day following tumor cell transplantation, and none of the mice were alive after receiving naive plasma. The use of convalescent plasma transfer in a clinical environment has been found to be successful, e.g., during the outbreak of the SARS-CoV-2 pandemic [74,75].

We are aware of the limitations of our model, such as (1) the relatively small number of animals/groups, (2) the lack of predictive markers for remission, and (3) the mechanistic investigation of adoptively transferred splenocytes. Several questions remain open, e.g., how to orchestrate adoptive transfer so as to achieve higher response rates. However, we developed a model system that can be used to activate tumor-reactive immune cells in non-immunosuppressive organisms, which is followed by applying activated tumor-reactive lymphocytes via adoptive transfer to treat 4T1 triple-negative breast cancer. Further analyses and development are needed in order to make this method of whole-cell vaccine preparation from tumor biopsy specimens more applicable, and to use it for vaccination preferably, but not exclusively, in HLA-matched tumor-free donors, followed by the transfer of tumor-reactive leukocytes and/or plasma to cancer patients. The advantages of our model include its lower cost and rapid availability when compared to personalized CAR-T or CAR NK-based immunotherapies. The proposed method of whole-cell vaccination makes a plethora of antigens available, without the need for difficult and expensive TSA or TAA identification [76]. 

## 5. Conclusions

The direct translation of the results of this study to clinical practice is beyond the scope of the current paper. However, our data may serve as a basis for future studies seeking to optimize conditions in a clinical setting, where a tumor biopsy is homogenized, attenuated, and used as a whole-cell vaccine administered to an HLA-matched non-tumor-bearing host (probably a voluntary family member). The sequence of whole-cell vaccine preparation from a tumor biopsy specimen should follow the microevolution of tumor mutational burden, and tumors should be kept under immunosurveillance. Following the development of tumor-reactive IgG antibodies, the transfer of plasma to cancer patient is suggested. The adoptive transfer of tumor-reactive T-cells requires thorough attention, and strict preliminary experiments should demonstrate how to avoid the graft-versus-host disease and avoid harm to patients. The authors do not want to overgeneralize the conclusions, and future studies are required using animal models to further develop the concept herein, whose results can then be combined with the outcomes of clinical trials to ensure safety and effectivity in a clinical setting.

## Data Availability

The raw data are available from the corresponding authors upon request.

## Supplementary Materials

The following supporting information can be downloaded at: https://www.mdpi.com/article/10.3390/vaccines11071254/s1, Figure S1: The individual 4T1 growth curves.
