# Supplementary material for: Introduction of an Ultraviolet C-Irradiated 4T1 Murine Breast Cancer Whole-Cell Vaccine Model
Source: Vaccines (Basel). 2023 Jul 18;11(7):1254. doi: 10.3390/vaccines11071254 (PMC10386199; doi:10.3390/vaccines11071254)

Supplementary Figure S1. The individual 4T1 tumor growth curves. Tumor growth curves of untreated (black line) or adoptively transferred (A, yellow line) bone marrow, (B, yellow line) plasma, or (C, yellow line) splenocyte injected animals. That Figure corresponds to Figure 3.

A

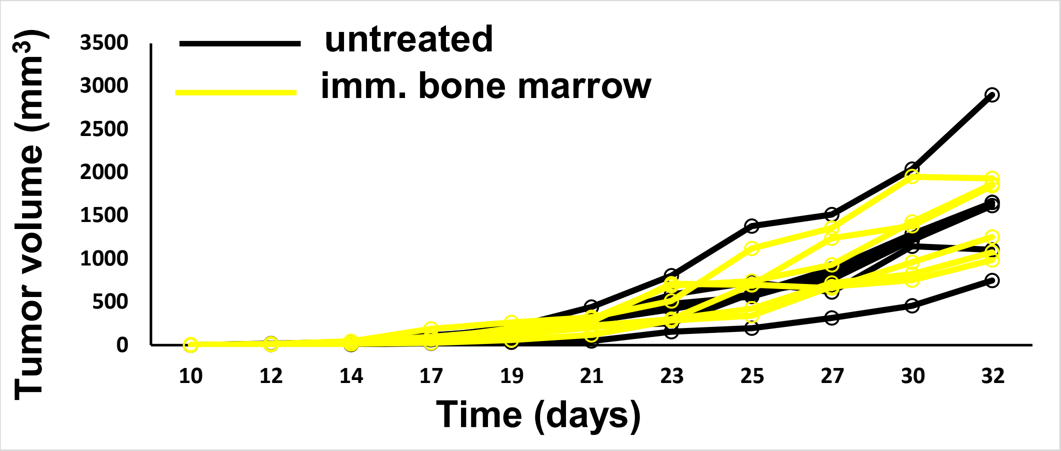

B

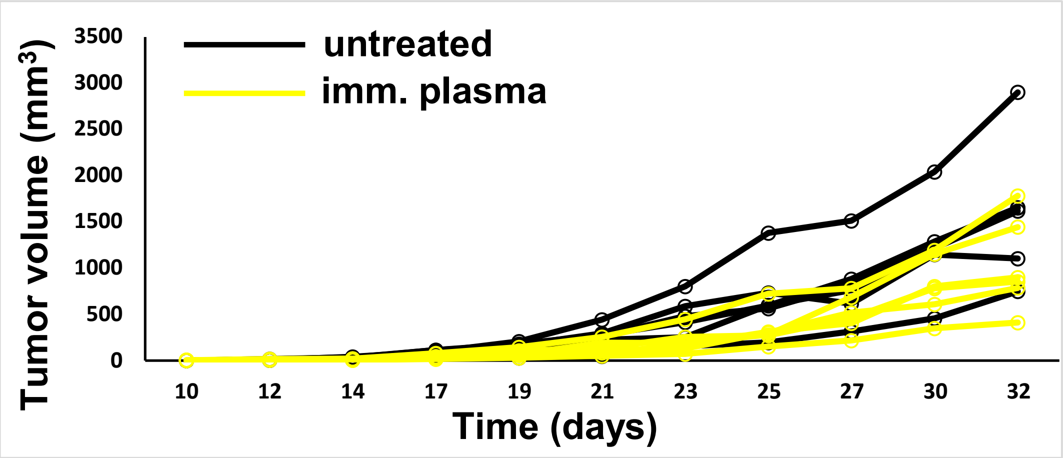

C

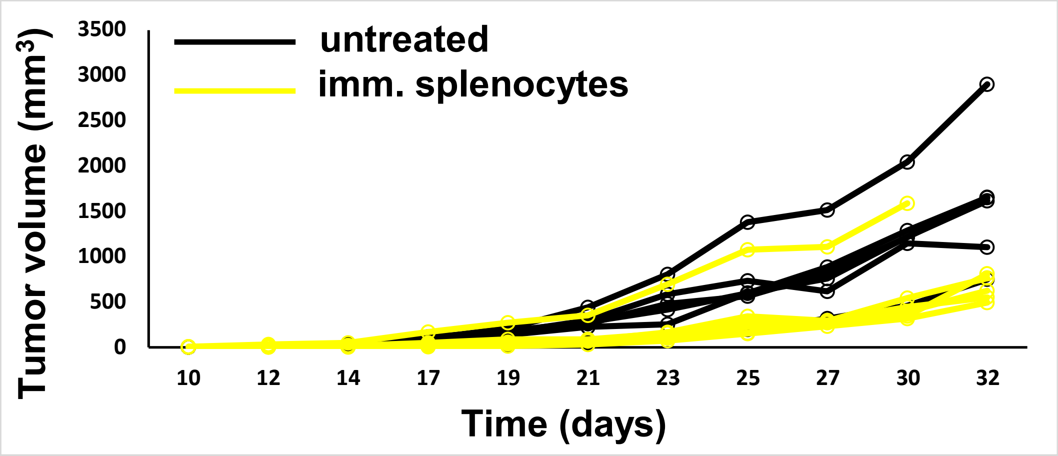

Supplement: Supplementary file 1 [file vaccines-11-01254-s001.zip › vaccines-2443148-supplementary.pdf]
